# Supplementary material for: Are carbon emissions trading and green financial instruments synergistic? -Comprehensive quantitative research based on content analysis
Source: PLoS One. 2024 Mar 7;19(3):e0298601. doi: 10.1371/journal.pone.0298601 (PMC10919601; doi:10.1371/journal.pone.0298601)
Supplement: S1 Data — (ZIP) [file pone.0298601.s001.zip › Data/地级市Z_F工作报告.zip!/╜¡╬≈/╙Ñ╠╢╩╨/└┤╫╘╣1⁄2╓┌║┼í╛┬φ┐╦╩2╛▌═°í┐_2008.docx]

各位代表：

　　　　现在，我代表市人民政府向大会作政府工作报告，请予审议，并请市政协委员和列席同志提出意见。

　　　　一、2007年政府工作回顾

　　　　刚刚过去的2007年，是本届政府的开局之年，是我市经济社会发展承前启后、继往开来的一年。一年来，在省委、省政府和市委的正确领导下，全市上下认真贯彻落实科学发展观，突出构建和谐社会这根主线，牢牢抓住超常发展这个关键，扎实推进“四大一新”发展战略，切实转变经济发展方式，与时俱进，开拓创新，圆满完成了市七届人大二次会议确定的各项目标任务，全市经济社会呈现又好又快发展的新态势。

　　　　——发展速度明显加快。预计全年完成生产总值206.49亿元，同比增长14%。规模以上工业主营业务收入780亿元，同比增长74.1%，其中地方规模以上工业主营业务收入260亿元，同比增长140.2%。实际完成财政总收入28.1亿元，同比增长43.3%，其中地方财政收入12.5亿元，同比增长46.8%。

　　　　——运行质量显著提高。三次产业结构为9.3：63：27.7，二产比重同比提高9.6个百分点。地方财政收入增幅历史上首次超过财政总收入增幅。财政收入占生产总值的比重达13.6%，税收占财政收入的比重达91.2%。市属规模以上工业增加值占全市规模以上工业增加值的比重达33.5%，同比提高19.9个百分点。

　　　　——发展后劲持续增强。全市预计完成全社会固定资产投资95亿元，增长30%，其中城镇固定资产投资77.2亿元，增长29.5%。共实施重点工程项目36项，累计完成投资58亿元。全年用电量达17.4亿度，增长23%。实现社会消费品零售总额49.48亿元，增长17.3%。投资和消费对经济增长的拉动力明显增强。

　　　　——县域经济不断壮大。贵溪市财政收入突破20亿元，连续五年位居全省第一。余江县、月湖区、龙虎山景区、鹰潭工业园区财政收入大幅度增长，其中鹰潭工业园区财政收入实现了翻两番的目标。全市财政收入过千万元的乡镇（街道）达13个，其中过亿元的乡镇有2个。

　　　　——节能减排成效显著。全面启动了绿色生态鹰潭建设，先后取缔了饮用水源一、二级保护区内的排污口。耕地总量保持总体平衡。空气和水质量分别达到国家二级和Ⅱ类标准。万元生产总值综合能耗同比下降6.1%，二氧化硫和化学需氧量全面完成年度减排指标。

　　　　——人民生活日益改善。城镇居民人均可支配收入预计可达11570元，增长19.9%；农民人均纯收入4406元，增长11%；城乡居民本外币储蓄存款余额108.5亿元，增长14.7%。省政府下达的80项民生指标基本完成，人民群众幸福指数显著增强。

　　　　一年来，我们紧紧围绕以推进“四大一新”来谋求经济社会超常发展的工作思路，着力抓了以下八个方面的工作：

　　　　（一）建设世界铜都取得阶段性成效，工业发展日趋强劲

　　　　按照省委、省政府提出的“鹰潭要建成世界铜都”这一战略定位，及时顺应铜产业集聚的新趋势，确立了以建设“三个基地、一个中心”为核心内容的发展目标，全市铜产业发展势头迅猛，铜产业实现主营业务收入700亿元、税收25亿元，其中地方铜企业实现主营业务收入237亿元、税收9.2亿元。在与江铜的战略合作上，双方签订了《共同发展铜产业壮大铜经济合作协议》，从发展目标、铜原料、铜加工、铜物流、铜文化等十个方面全面加强合作。在铜冶炼基地建设上，贵冶四期技改扩建顺利投产，标志着江铜以70万吨电解铜生产能力，跻身世界铜行业前三强。在铜废旧原料再生利用基地建设上，通过招商引资新增阳极板、粗铜锭等铜原料60万吨，为江铜和本地铜加工企业发展提供了原料保障；铜产业循环基地拆解加工区已获国家环保总局批准，成为我国内陆地区唯一的铜拆解加工区；由国内最大的废旧铜回收利用企业广东南海陈氏集团投资建设的铜拆解加工区项目进展顺利，一期1000亩土地平整已基本完成。在铜产品加工基地建设上，全国500强企业浙江金田、国内铜板带加工前三强宁波兴业等一批规模以上铜加工企业相继落户我市，其中金田铜业实现了当年开工、当年投产、当年预计可完成主营业务收入35亿元。全市铜加工能力达116万吨，新增50万吨，铜加工企业达65家，新增28家，初步形成了粗铜、电解铜、铜杆、铜线、铜管、铜棒、铜板带、异型材及铜铸件等上下游产品配套的铜产业链。

　　　　在铜产业快速发展的同时，其他工业产业的发展也保持了强劲的势头，水表、节能灯、眼镜、微型元件、医药、食品、轻纺、化工等地方特色产业的发展都出现了喜人的局面。在重点产业的强力拉动下，全市工业经济快速发展，规模以上工业增加值达124亿元，增长21%，其中市属规模以上工业增加值达41.5亿元，增长57.5%。鹰潭、贵溪两个工业园区主营业务收入均突破100亿元，预计分别可达105亿元和115.8亿元，双双跻身全省超百亿十强工业园区之列。余江工业园区预计可实现主营业务收入20.5亿元，增长20.5%。贵溪铜城220千伏、鹰潭工业园区110千伏、余江五湖110千伏变电站相继竣工投运，为我市工业经济又好又快发展奠定了扎实基础。

　　　　（二）打造现代物流中心开局良好，区位交通优势日趋凸显

　　　　全市物流产业呈现呼之欲出的发展态势，以运起步初见成效，全年新增货运车辆820辆、吨位5746吨。货运车辆外挂流失现象得到基本扭转，先后引进了中联物流等5家大型物流企业，开通了3条省际物流专线，认定了9家规模以上汽车货运企业，其中江铜物流公司实现营业收入1.5亿元，上缴税费1400万元。全面加强了与宁波港集团、中国诚通集团、中铁现代物流、中国邮政物流等大型物流龙头企业的沟通合作，与宁波港集团签订了合作建设“无水港”项目协议，中国邮政物流公司将鹰潭列入全国邮政物流节点城市。

（七）社会事业明显进步，经济社会发展日趋协调

国家新材料产业化示范基地建设快速推进。组织申报了32个国家和省级科技项目，2家企业被批准为国家高新技术企业，3个项目被成功列入省科技成果产业化项目。各级各类教育均衡发展，教育教学质量不断提高，素质教育全面推进，我市参加第八届全国中小学电脑机器人竞赛获得冠军；国家二类城市语言文字工作得到省评估团的充分肯定；改造中小学校危房2.2万平方米。文化事业多姿多彩。两首原创歌曲获全省“五个一工程”奖。广播电视“村村通”覆盖率进一步提高。城市社区卫生服务发展迅速，计划免疫工作全面达标，重大疾病防治和卫生执法监督得到进一步加强。强化了市场监管，组织开展了产品质量和食品安全专项整治行动。加大了整治性别比失调工作力度，计划生育率为89.36%，人口自然增长率控制在7.85‰以内。组团参加了江西省第三届全民健身运动会，荣获团体总分一等奖。成功举办了全国女篮俱乐部联赛鹰潭赛区比赛。进一步落实了拥军优属政策和优惠措施，开展了以庆祝建军80周年为主题的一系列纪念活动，广大驻鹰部队官兵和民兵预备役人员为建设和谐平安鹰潭做了大量卓有成效的工作。国土、审计、人事编制、档案、人防、新闻出版、民族宗教、外事侨务、气象服务、防震减灾、老龄、残疾人等各项工作健康协调发展。

（八）改善民生成效显著，社会环境日趋和谐

民生工程普遍惠民。围绕 “四个全覆盖”、“四个倾斜”，共安排1.23亿元财政配套资金，用于发展民生工程。城镇新增就业2.3万人，零就业家庭就业安置率达100%，“4050”人员就业1500 人，发放小额担保贷款1.34亿元。发放养老金1.72亿元，人均月基本养老金水平达734元。发放低保金4010万元，城乡低保人均月补差水平分别达90.36元和33.18元。新型农村合作医疗参合人数64.2万人，参合率达90.7%，在全省排位第一。城镇居民基本医疗保险参保人数达6.2万人。为全市公办义务教育阶段学生免除杂费2754万元，为贫困家庭学生免教科书费227万元，为7015名贫困家庭学生补助寄宿生生活费216万元，资助普通高中贫困生2131人，资助高考贫困生356人，资助中职学校贫困生13502人，资助贫困大学生977人，全年共拨付义务教育公用经费1170万元。经济适用住房和谐小区一期工程顺利竣工，二期工程全面开工，廉租住房受益户208户。社会环境更趋稳定。坚持依法行政，规范权力运行，政务环境进一步优化。“五五”普法成效明显，依法治市战略不断深入。城乡治安防控体系建设稳步推进，严厉打击了各类违法犯罪活动。深入开展了矛盾纠纷大排查，严格落实了“四定四包、四个决不放过”责任制，社会政治保持总体稳定。生产安全、食品药品安全、交通安全和消防安全态势总体平稳。

各位代表，过去的一年，是我市经济社会步入又好又快发展轨道的一年，是经济活力和后劲大为增强的一年，也是城乡居民受益最大、得实惠最多的一年。一年来，我们所取得的成绩，是省委、省政府和市委正确领导的结果，是全市人民同心同德、扎实苦干的结果，是人大依法监督、政协民主监督的结果，是各民主党派、工商联、群众团体和驻鹰单位、人民解放军、武警官兵以及离退休老同志共同努力和大力支持的结果。在此，我代表市人民政府，向全市人民，向所有关心、支持鹰潭经济社会发展的各界人士，表示衷心的感谢并致以崇高的敬意！

在肯定成绩的同时，必须清醒看到，我们的工作离省委、省政府和市委的要求还有不少差距，离人民群众的期望还有一定距离，主要表现在：经济总量仍然偏小，运行质量有待进一步提升；县域经济发展不够平衡，潜力和后劲有待进一步挖掘；统筹城乡协调发展、节能减排的任务依然十分艰巨；城市功能不够健全，管理水平尚待提高；行政效能还需进一步优化。这些问题必须引起我们的高度重视，采取切实有效的措施，认真加以解决。

二、2008年政府工作的总体要求和预期目标

今年，是深入贯彻党的十七大精神的第一年，也是谋求鹰潭经济社会超常发展的关键一年，做好今年的政府工作，意义十分重大。今年政府工作的总体要求是：高举中国特色社会主义伟大旗帜，以邓小平理论和“三个代表”重要思想为指导，深入贯彻党的十七大、中央经济工作会议和省委十二届四次全会、市委六届四次全会精神，全面落实科学发展观，加快转变经济发展方式，积极推进改革开放和自主创新，精心实施“四大一新”发展战略，统筹城乡协调发展，高度重视改善民生，抢抓机遇，敢于崛起，善于跨越，积极赶超，着力构建绿色生态鹰潭、创新创业鹰潭、和谐平安鹰潭，努力打造独具鹰潭特色的经济“小巨人”。

2008年主要预期目标是：全市生产总值242亿元，增长13%；财政总收入32.5亿元，增长15.5%，其中地方财政收入14.5亿元，增长15.5%；全社会固定资产投资116亿元，增长22%；社会消费品零售总额58.5亿元，增长18%；城镇居民人均可支配收入12610元，增长9%；农民人均纯收入4706元，增长7%；城镇就业率达94.5%，居民消费价格指数上涨幅度控制在4-5%以内，人口自然增长率控制在8‰以内；万元生产总值综合能耗下降4%，化学需氧量和二氧化硫排放总量控制在省下达的目标以内。

实现上述目标，政府工作在指导思想上必须牢牢把握以下原则：一要坚持好字优先，科学发展。把科学发展观贯穿于各项工作的始终，积极策应宏观调控对优化经济结构、提高运行质量提出的新要求，切实把防止经济增长由偏快转为过热、防止价格由结构性上涨演变为明显通货膨胀作为宏观调控的首要任务，按照控总量、稳物价、调结构、促平衡的基调做好宏观调控工作,努力实现经济社会又好又快发展。二要坚持改善民生，促进和谐。从人民群众最关心、最直接、最现实的利益问题入手，千方百计增加城乡居民收入、扩大社会保障、解决困难群众基本生活、满足人们精神文化需求、维护社会安全稳定，切实做到发展为了人民、发展依靠人民、发展成果由人民共享。三要坚持工业带动，就业为重。始终把推进新型工业化摆在经济工作的突出位置，依靠工业的大发展，更有力地支持和反哺新农村建设，支撑和推进城市化进程，增强政府的公共服务和社会管理能力，着力使经济增长带来更多的就业岗位，惠及广大的人民群众。四要坚持强化基础，狠抓落实。切实加强基层基础工作，充分发挥基础产业的带动作用、基础设施的支撑作用、基础工作的保障作用。聚精会神抓好各项工作的落实，注重从理解、操作、执行、落实层面研究解决具体问题，建立抓落实的促进和保障机制，形成抓落实的综合考核评价体系。

三、2008年政府主要工作任务和措施

（一）咬定超常发展，奋力实现全市经济好中求快

1.坚持以建设世界铜都为龙头，力促工业经济实现新跨越。突出以江铜为依托，以园区为载体，以循环经济为主线，以科技创新为动力，不断提升铜产业集聚能力，力争实现阴极铜生产能力90万吨，铜加工能力160万吨，铜产业主营业务收入800亿元、税收30亿元。扎实推进铜拆解加工区建设，明确项目法人和责任主体，理顺管理模式和运作办法，尽快形成国内和国外供货能力，确保拆解加工区监管中心和污水处理厂建成使用，力争年内有10家以上拆解企业建成投产，形成30—50万吨拆解能力。按照“上下延伸、双向扩张”的思路，加大铜产业招商力度，积极争取江铜集团公司帮助引进2—3个投资过亿元的铜加工项目，力争引进1—2家世界500强和国内300强铜企业，确保全年铜加工企业突破100家。全面深化与江铜集团公司的战略合作伙伴关系，健全完善实质性合作推进机制，共同争取上海期货交易所在鹰潭设立铜期货交割库，全力支持贵冶电解车间东扩改造工程，积极配合江铜集团公司整合铅锌资源。高起点编制铜产业循环基地总体规划和控制性详规，争取列入国家和省循环经济试点。进一步完善建设世界铜都发展规划。积极开展铜产业技术攻关，加快建立国家铜冶炼及加工工程技术中心和铜科技孵化器，为世界铜都建设提供科技支撑。倾力支持贵电“上大压小”技改工程，力争年内开工建设。继续精心组织实施产业经济“十百千亿工程”，大力扶持眼镜、微型元件、节能灯、水表、雕刻、中医药、食品、服饰等特色优势产业，培植1—2个主营业务收入超过100亿元的地方龙头企业，确保全市规模以上工业实现主营业务收入900亿元。

2.坚持以交通区位优势为依托，力促物流产业发展取得新突破。大力发展现代运输。突出以运起步，优化物流资源配置，力争培育规模以上货运企业20家以上，营业收入过千万元、利税过百万元的企业10家以上，确保年内完成货运车辆运力3.15万吨，努力将汽车货运产业发展成为主营业务收入超5亿元、对地方财政贡献超5000万元的特色产业群。全面推进物流合作。全面深化与宁波港集团的合作，开工建设“无水港”项目。积极跟进和落实与中国诚通集团、中铁现代物流、中国邮政物流等国内物流龙头企业达成的合作协议和意向，力促城市物流中心、汽车贸易中心、铜现货市场等项目取得突破性进展，努力把鹰潭打造成全国区域性现代物流中心。积极发展仓储、批发、流通加工等产业，推进物流业全面发展。积极开展物流招商。进一步完善促进汽车货运业发展的政策措施，鼓励和吸纳外地企业来我市创办汽车运输公司，大力吸引沿海发达地区客商来鹰投资创办物流企业，力争全年引进2家以上国内外知名物流企业来我市建设物流基地。精心编制物流规划。加强与国家发改委宏观经济研究院、北京邮电大学的合作，全面完成“十一五”物流发展规划，以规划指导建设，确保各项工作有序开展。

3.坚持以做旺旅游为重点，力促第三产业再现新繁荣。以打造“精品景区、休闲城市、魅力乡村、和谐旅游”为切入点，着力发展大旅游，积极开拓大市场，努力形成大产业，确保全年接待国内旅游者770万人次，接待境外旅游者6.6万人次，旅游总收入和旅游创汇分别增长26.8%和21%，其中龙虎山核心景区接待游客40万人。精心打造品牌。用好“世界地质公园”、“中国优秀旅游城市”、“中国旅游竞争力百强城市”等金字招牌，深入推进龙虎山创建国家5A级旅游区、联合申报世界自然遗产工作，力争龙虎山创建国家5A级旅游区获得通过，联合申报世界自然遗产取得实质性进展。完善旅游要素。大力培植“吃、住、行、游、娱、购”相衔接的旅游产业，进一步调整优化旅游线路，完善龙虎山景区门票体制，理顺道教活动场所管理体制机制，实现由门票经济向旅游产业经济转变，由半日游向1—2日游转变，由单一观光型向观光、休闲、度假、会议、养生综合型转变。积极引进国内外知名旅游集团参与我市旅游要素开发。科学整合县（市、区）旅游资源，延伸旅游产业链，着力构建大旅游格局。切实加快旅游星级宾馆和相关配套设施建设步伐，开工建设龙虎山新景门、游客服务中心、大型生态停车场，力争今年全市旅行社达45家、星级宾馆达25家、旅游直接从业人员达1.5万人。加大宣传促销。突出打好“华夏道都”、“绝特山水”牌，加大鹰潭国际旅游整体形象的宣传推广力度，集中力量拓展港澳台、东南亚、韩国、日本客源市场，进一步做旺人气，提升鹰潭龙虎山知名度。发展乡村旅游。结合社会主义新农村建设，着力发展景区带动型、城郊休闲型、生态观光型、民俗风情型等乡村旅游模式，培育一批民俗风情浓郁的农家旅馆，开发一批具有地方特色的生态景点，建设一批集游览观光、休闲度假、登山健身等功能于一体的乡村旅游胜地。通过上述举措，进一步让鹰潭旅游名气响起来，人气旺起来，效益好起来。大力发展总部经济、楼宇经济和结算经济等现代服务业，全面推动第三产业整体跃上新水平。

4.坚持以提高资本运作水平为纽带，力促经济发展增添新活力。充分发挥资本运作对加快经济发展的“助推器”作用，积极借助资本市场的“输血”功能培植壮大地方企业的“造血”功能。按照“政府推动、多方参与、点面结合、市场孕育”的原则，扶持引导成长性强的企业上市融资，重点抓好天施康药业、三川水表、南方照明等企业的上市工作，全力支持兴业电子、世纪阳光照明等上市企业在我市的生产基地做大做强，扎实做好宏磊铜业、晨宇铝业等上市后备企业的规划完善工作，积极培育一批上市后备资源，打造一批上市后备梯队。科学整合政府性资产、资本、资金和资源，进一步理顺城市开发、经营、管理体制，完善城市开发项目市场化运作机制，拓宽城市开发项目多元化融资渠道，规范土地收、储、供制度，扎实推动城市资本的高效、健康、协调运作，不断提高城市资本运作水平。

5.坚持以招大引强为主线，力促开放型经济跃上新台阶。改革开放是实现鹰潭超常发展的关键抉择。过去发展靠改革开放，今后实现新跨越更要靠改革开放。没有改革就没有活力，没有开放就没有动力。必须坚定不移地实施大开放主战略，依托工业园区，瞄准世界500强、国内300强、行业20强企业，大力引进名牌、名企项目，力争在引进内资单项投资超10亿元、外资单项投资超5000万美元的大产业和大项目上有新突破，力争年内每个工业园区引进2家以上固定资产投资过亿元、主营业务收入超10亿元的企业，确保全年5000万元以上省外工业项目实际进资31亿元，实际利用外资8600万美元。加快园区发展。进一步加大工业园区基础设施建设力度，优化园区发展环境，充分利用企业纳税大户奖励办法、工业企业发展奖励办法等激励措施，促进工业向园区集中、生产要素向优势企业聚集，鹰潭工业园区、贵溪工业园区主营业务收入要再上新台阶。积极筹建宁波工业园。突出产业招商。依托我市具有比较优势的特色和支柱产业，积极顺应沿海产业转移的新趋势，努力引进一批产业核心项目，延伸产业链条，壮大产业集群，着力提高引进项目的投资总量、科技含量、纳税数量、就业容量。创新招商方式。认真落实推进招商引资工作、加快工业经济发展的一系列政策措施，充分发挥协同联动招商机制的作用，大力开展以商招商、以情招商、以诚招商，吸引更多的投资者来我市投资兴业。提升招商实效。对有投资意向的项目，落实专门班子和人员粘紧盯牢，促其尽快签约；对已签约项目，实行跟踪问效，促其早进资、早开工；对重点在建项目，加强协调调度，提供全方位服务，促其按时竣工、早日投产；对已投产项目，协调帮助企业解决生产经营中遇到的困难和问题，促其开足马力，尽快达产达标。做大外贸总量。进一步转变外贸增长方式，优化产品结构，提高机电产品和高新技术产品的出口比重，加快口岸建设，优化通关模式，降低通关成本，壮大我市自营出口企业队伍，确保全市外贸出口达9600万美元，力争突破1亿美元。

（二）立足协调发展，努力提高经济社会的可持续发展能力

1.大力推进城市化进程，努力提升城市品位。坚持以规划为龙头，以建设为重点，以管理为手段，全面落实优先发展城市公交战略，加快城市畅通工程建设步伐，进一步拉开城市框架，高品位建设以信江为轴、以两岸自然风貌为依托、以城际快速通道为纽带的“显山、露水、见城、滨江”生态宜居城市。科学编制城市规划。按照城市人文景观与自然景观和谐统一、人工系统与自然系统相互交融的城市规划理念，精心做好鹰潭市城市总体规划的修编，启动鹰西片区、夏埠片区、雄鹰大道两侧控制性详规的编制。强力推进城市建设。重点抓好20件实事：（1）完成雄鹰大道建设；（2）建成市体育场；（3）建成市青少年活动中心；（4）完成龙虎山大道延伸工程；（5）完成胜利西路延伸工程；（6）完成五洲路延伸工程并着手开展鹰西大道前期工作；（7）完成府前路延伸工程；（8）完成南站路综合改造；（9）建成城市生活污水处理厂，开工建设鹰潭工业园区污水处理厂；（10）建成环境监控指挥中心；（11）实施一批小街小巷硬化亮化工程；（12）开工建设206国道天禄至景区段公路改造工程；（13）开工建设鹰瑞高速公路龙虎山连接线；（14）开展鹰西湿地公园前期工作；（15）建设8.5万平方米经济适用住房和126套廉租住房；（16）开工建设老干部活动中心；（17）开工建设市科技馆；（18）启动沿江小区旧城改造；（19）启动铁路生活片区综合改造；（20）启动市行政服务中心建设。切实加强城市管理。继续深入开展“迎省运会、创文明城”活动，巩固城市管理综合执法、客运市场秩序整顿、联建共创文明社区成果，加大市容环境综合整治力度，突出抓好“城中村”和城乡结合部改造，大力整治违法建筑。进一步理顺城市管理体制机制，健全完善城市管理综合执法职能，切实提高交警、城管、环卫三支专业队伍素质，积极探索创新城市管理长效机制。

2.高度重视“三农”工作，大力推进新农村建设。以促进农业增产、农民增收为目标，以社会主义新农村建设为抓手，稳定粮食生产，优化产业结构，做特农业经济。推进新农村建设。全面完成200个左右新农村建设试点村任务，努力打造一批可学可看可推的新农村样板，实现我市新农村建设在全省站前列的目标。落实惠农政策。扎实做好财政惠农补贴资金“一卡通”发放工作，确保各项补贴资金实实在在发到农民手中。稳定粮食生产，确保粮食种植面积150万亩以上、粮食总产55万吨以上。加强农副产品价格监控，扎实做好能繁母猪饲养补贴和政策性保险工作。加快产业化进程。培植壮大南方早熟梨、笋竹、良种猪、花卉苗木、板栗、花生六个主导产业，扶持发展蔬菜、中药材、有机茶、油茶、水产、木（竹）精深加工等特色产业。用活用好农业产业化扶持资金，力争年内培植壮大2个年销售收入超亿元、4个年销售收入超5000万元、10个年销售收入超1000万元的龙头企业以及1—2个条件成熟的农民专业合作组织。加强农业农村基础设施建设。加快农村公路建设，完成农村公路硬化200公里，实现全市100%的行政村通水泥（油）路。加快城乡公交一体化进程，新建120个行政村客车招呼站、17个乡镇客运站，建设渡改桥10座。继续深入推进造地增粮富民工程。打好病险水库除险加固攻坚战，确保汛前基本完成省规划二期128座病险水库除险加固任务。扎实推进城市防洪日元贷款项目建设，开工建设东湖沿江防洪堤，加大万亩以上圩堤除险加固力度。改善农村生产生活条件，解决2万农村人口饮水安全问题，做好余江白塔渠灌区水利血防续建工程。以平原造林绿化为重点，推进血防林、长防林、公益林等重点林业工程建设。加快园田化、土地整理、农田林网等工程建设，打造园田化建设示范区。进一步完善防汛抗旱、森林防火、动物防疫三大防控体系建设。

3.突出扩大总量，做强县域经济。激发县域发展活力。按照“事权下放、重心下移、责任明晰”的原则，进一步清理和规范行政审批事项，简化审批手续，赋予县（市、区）更大的自主发展权，为县域经济发展营造更加宽松的环境。凸显县域发展特色。引导各县（市、区）因地制宜，扬优成势，筛选1-2个能充分发挥县域资源、品牌、人文、区位优势的主导产业，按照区域化布局、专业化生产、规模化经营的要求，集中力量培育一批特色产业群，努力形成各具特色的县域产业发展新格局。大力发展乡镇经济。积极引导各乡镇依托工业园区发展工业、依托驻地企业发展配套产业、依托本地优势农产品发展加工业，大力推广樟坪经验，鼓励乡镇把项目引进落户到园区发展，逐步做大乡镇财政蛋糕，力争年内财政收入过亿元的乡镇达3个，过千万元的乡镇达16个。强化考核机制。科学制定县域经济考核评价体系，实行平时动态管理与年度综合考核相结合，加大对县域经济发展的考核奖励力度，安排专项资金，对县域经济发展快的县（市、区），对经营效益好、纳税多的纳税大户，对支持县域经济发展、信贷投放增幅大的金融机构，对发展速度快、财政增幅高的乡镇给予奖励，推动各地迅速掀起“敢于崛起，善于跨越，积极赶超”的热潮。

4.鼓励支持全民创业，做活城市经济。实践充分证明，推进全民创业，可以使经济发展快起来，人民生活富起来，体制机制活起来，社会关系和起来。各级政府要充分调动人民群众的创业热情，使创业光荣、创业有功成为全社会的主流思想，使自强不息、勇创一流成为创业者的价值追求，使助你成功、成就创业成为全社会的道德风尚，使开拓创新、服务创业成为政府各部门的核心理念，让人民群众的创业愿望得到尊重，创业活动得到支持，创业才能得到发挥，创业成果得到肯定。今年，政府要从以下几方面努力做好工作，支持全民创业。搭建创业平台。高标准、高质量建设全民创业园，力争用两年时间建成集创业、办公、商住为一体的创业发展平台。扎实做好乡镇创业园和中小企业孵化器基地的规划论证工作。扶持创业主体。设立全民创业专项资金，对市场前景好、潜力大、后劲足的创业项目给予重点扶持，对在全民创业中涌现出的本土创业典型给予一定奖励。组建全民创业担保机构，鼓励民间资本参与融资担保，积极为符合条件的本土企业提供信用担保。开通鹰潭创业网，为各类创业主体提供创业信息服务。安排专项经费，加大对创业人员的培训力度，大力培育本土创业主体。落实创业措施。大力实施就业创业工程、回乡创业工程、中小企业成长工程、创业培训工程，鼓励各类人员自主创业，支持各类企业二次创业，全面落实扶持全民创业的各项政策措施，切实优化全民创业的政策环境、政务环境、法制环境、人文环境，着力提高全民创业对财政的贡献率。做活城市经济。充分利用现有城市资源，搭建交易平台，吸引有实力、讲诚信的投资者参与城市经济建设，进一步增强经济发展的内生动力，努力实现内外源经济协调联动发展。

5. 深化各项改革，创新体制机制。深入推进农村综合改革，着力抓好林业产权制度配套改革，全面深化粮食流通体制改革，积极稳妥地推进国有农场税费改革。进一步推进国库集中支付改革，扎实做好财政惠农资金制度和支付方式“一卡通”改革工作。继续实施好收入分配制度改革，全面完成参照公务员管理人员的工资套改。创新机制，积极探索适应新形势的拆迁安置办法。深化国有资产监督管理体制改革，搭建国有资产融资平台。稳步推进金融体制改革，大力支持国有商业银行体制改革和邮政储蓄体制改革，加快组建村镇银行，建立民间担保机构，健全联保互助体系，逐步破解中小企业贷款难问题。

（三）围绕和谐发展，让人民群众共享改革发展成果

1.深入推进民生工程。把改善民生作为建设和谐社会的首要任务，最大限度地提高人民群众的幸福指数。突出重点，积极推进就业再就业。大力发展面向企业的生产性服务业、面向居民的生活性服务业、面向社会的公共性服务业，全面落实促进就业优惠政策，进一步扩大小额贷款发放规模，建立健全市、县、乡一体化公共就业服务网络，加大城镇职业培训和农村劳动力转移培训力度，提高培训质量和就业层次，确保全年新增城镇就业1.96万人，新增转移农村劳动力1.1万人。打造亮点，全面提升医疗保障水平。实现新型农村合作医疗全覆盖，积极推行市、县、乡三级定点医疗机构直补工作；提高城镇居民基本医疗保险参保面，力争参保人数达8.8万人。扩大医疗救助覆盖面，提高医疗救助资金使用率，确保城乡医疗救助资金使用率达80%以上。突破难点，着力提高社会保障覆盖面。进一步完善社会保险扩面征缴目标责任体系和社会保险基金损失责任追究制度，建立适合农民工和被征地农民特点的养老保险制度，妥善解决未参保城镇大集体困难企业退休人员生活困难问题，力争用两年左右的时间实现园区企业参加养老保险全覆盖，确保养老保险基金扩面征缴总额达1.45亿元。提高城乡低保水平，确保城市低保对象人均月补差达95元以上，农村低保对象人均月补差达35元以上。关注热点，努力实现学有所教、住有所居。巩固“两基”成果，健全义务教育经费保障机制，全面免除城乡义务教育阶段中小学生课本费。完善高、中等职业学校家庭困难学生资助政策体系，继续对考入大学的特困家庭学生实行补助。扎实推进经济适用住房和谐小区二期工程建设，千方百计增加廉租住房房源，逐步实现中低收入家庭住有所居。

2.努力实现生态文明。大力发展循环经济，把节能减排、保护环境、集约用地作为转变经济发展方式、促进科学发展的重要抓手，精心打造“一流的空气、一流的水质、一流的生态、一流的居住环境、一流的绿色生态保护建设管理机制”。加大重点领域、重点行业、重点企业的节能力度，突出抓好铜加工、医药、电力、建材化工等重点耗能行业的节能工作，认真实施“十大重点节能工程”，树立一批节能示范企业。切实加强我市列入国家千家企业节能行动和全省百户重点耗能企业的四家企业的节能监督工作。扎实推进建筑节能，着力加强交通节能，大力倡导民用节能。积极推进清洁生产，强化绿色、无公害产品认证和绿色标志管理。坚持“既要金山银山，更要绿水青山”，深入实施环保“十大工程”和“七项专项整治行动”，全面推进绿色生态鹰潭建设。强化污染物排放总量控制，加大农村污染治理力度，继续抓好全市饮用水源保护。严格保护耕地，切实做好全市土地利用总体规划修编和全国第二次土地调查工作，认真执行土地利用年度计划，控制总量，盘活存量，节约集约用地。

3.统筹发展社会事业。积极推进创新型鹰潭建设，切实加快全国科技进步示范市和国家新材料产业化示范基地建设步伐。实施一批科技成果集成转化重大科技专项。全面加快教育、文化、医疗卫生、体育产业发展。坚持教育优先发展战略，科学整合教育资源，促进城乡教育均衡发展。全面推进素质教育，扎实推进普通高中新课程改革，努力提升教育教学质量。大力发展具有鹰潭特色的职业教育，提高教育服务经济建设的能力。加大公益性文化设施建设投入，实施乡镇文化站建设工程。继续开展非物质文化遗产普查和第三次全国文物普查工作。加快广播电视数字化进程和农村广播电视村村通建设步伐，进一步完善公共文化服务体系。加强文化市场和新闻出版市场监管，深入开展“扫黄打非”，繁荣净化文化市场。切实加强城市社区卫生工作，完善社区卫生服务功能，扎实做好社区公共卫生服务券发放管理工作。优化城乡卫生资源配置，积极开展公立医院支援基层医疗服务工作，着力改善基层卫生机构基础设施条件。加强公共卫生体系建设，不断完善疾病控制、卫生监督、医疗救助服务功能。继续深化农村药品“两网”建设，整顿和规范药品市场秩序。围绕“迎奥运，备省运”，精心做好承办第十三届省运会的各项筹备工作。大力开展全民健身运动，着力提高竞技体育水平。严厉打击“两非”，加大出生人口性别比失调整治力度，健全计生利益导向机制，切实稳定低生育水平。做好军转干部安置工作。认真做好民兵预备役和人防工作，大力支持军队和国防建设。统筹做好审计、统计、物价、质监、气象、民族宗教、外事侨务、涉台事务、档案史志、妇女儿童、老龄、残疾人等工作。

4.切实维护社会稳定。深入开展法制宣传教育，弘扬法治精神，形成自觉学法、守法、用法的社会氛围。正确处理人民内部矛盾，探索建立科学有效的利益协调机制、矛盾调处机制、权益保障机制，最大限度地增加和谐因素，最大限度地消除不和谐因素。强化信访工作责任制，拓宽社情民意表达渠道，实现群众利益诉求制度化、规范化、法制化。完善突发事件应急管理体制。加强社会治安防控体系建设，依法严厉打击各类违法犯罪，切实增强公众安全感。强化安全生产责任制，实施重大危险源监控和重大事故隐患排查，切实抓好非煤矿山、烟花爆竹、道路交通、娱乐场所、食品等重点行业和领域的安全专项治理，确保安全态势总体平衡。

四、加强政府自身建设，塑造人民政府新形象

进一步转变政府职能，推动政府管理创新，着力提高政府的亲和力、决策力、执行力和公信力，努力打造勤政为民、改革创新、求真务实、诚信守法的政府。

（一）勤政为民，着力提高政府亲和力

坚持党的群众路线,做到思想上尊重群众、行动上深入群众、工作上依靠群众、生活上贴近群众，恪守为民之责，善谋富民之策，多兴利民之举，大办亲民之事。进一步转变政府职能，强化社会管理、公共服务，努力为群众提供更多、更优、更加均衡的公共服务产品，不断增强人民群众的幸福感。进一步规范行政行为，把为民谋利作为工作的出发点和落脚点，为群众真心实意做好事，千方百计解难事，以出色的政绩造福于民。进一步拓宽联系群众的渠道，扩大政府信息公开范围，保障群众对政府工作的知情权、参与权、监督权。高度重视信访工作，使群众来信来访成为政府了解民意、解决民忧、维护群众切身利益的重要渠道，让群众有机会讲话、有地方讲理、有人帮助解决问题。关心困难群众的生产生活，使他们切实感受到党和政府的关怀和温暖。

（二）改革创新，着力提高政府决策力

坚持解放思想、与时俱进，努力使政府的各项决策体现时代性、具有前瞻性、富于创造性。大力弘扬改革创新精神，把改革创新贯穿于政府工作的每一个环节，不断谋划新思路、推出新举措、开创新局面。健全重大事项决策的规则和程序，规范重大事项决策的提出、讨论、实施、反馈纠编和责任追究机制，对因决策失误给国家和人民群众利益造成重大损失的，要严格依法追究责任。建立健全公众参与、专家认证、集体决策相结合的决策机制，凡是涉及经济社会发展的重大决策，都要坚持调查研究和集体决策；凡是政策性、业务性强的事项，都要吸收有关方面的专家参与评议，并对实施效果进行评估；凡是与人民群众利益密切相关的重大事项，都要坚持实行社会公示或听证，真正做到重大决策合乎规律而定，工作举措顺应民心而生。

（三）求真务实，着力提高政府执行力

大兴求真务实之风，力促公务人员真正把精力集中到想干事上，把本领体现到会干事上，把目标锁定到干成事上。增强执行的自觉性，提高落实的主动性，在政府机关中努力形成政令通、节奏快、效率高的局面。进一步理顺部门分工，合理界定权责，解决部门职能错位、缺位、越位的问题，形成良好的政务运行机制。加强政府行为规范化建设，严格按规章制度和法定程序办事，力戒随意性；进一步精简行政审批事项，简化审批程序；积极探索行政综合执法改革，相对集中行政处罚权；建立工作责任机制，形成千斤重担人人挑的局面；建立工作奖罚激励机制，完善政绩考核评估体系，把求实效、创业绩作为能力和才干的鉴别标准。要把提升软实力、优化投资创业环境作为推动经济社会又好又快发展的关键举措来抓，深入开展政务环境优化年活动，加强政府部门效能建设，建立健全科学的机关效能量化、考核评估和责任追究机制，激励多干事，奖励干成事，治理不作为，惩戒乱作为，力促全市上下工作作风明显改进，服务能力明显增强，办事效率明显提高，发展环境明显优化。

（四）诚信守法，着力提高政府公信力

大力推进诚信政府建设，努力保持政策的连续性和稳定性，切实做到言必信、行必果。增强公仆意识，加强公务人员的思想观念和道德建设，建立公务人员的道德责任制度，强化公务人员做人的良心、做事的决心、做官的公心教育，培养其忠于民、信于民的优秀品质。增强大局意识，坚持依法行政，坚决反对行政权力部门化、部门权力利益化，做到令行禁止，保证政令畅通，提高政府工作质量和效率，切实解决有法不依、执法不严和多头执法等群众反应强烈的问题。增强节俭意识，坚决制止铺张浪费，努力降低行政成本，建设节约型政府。坚持廉洁从政，切实纠正行业不正之风，严肃查处违法违纪案件，不断增强各级干部的“免疫力”，努力打造人民满意的政府新形象。

各位代表，形势令人鼓舞，机遇催人奋进。让我们高举中国特色社会主义伟大旗帜，紧密团结在以胡锦涛同志为总书记的党中央周围，在市委的正确领导下，团结和依靠全市人民，心无旁骛谋事业，凝心聚力促发展，不辱使命，不负重托，为实现鹰潭经济社会科学发展、和谐发展、超常发展而努力奋斗！
